# A successful defence strategy in grapevine cultivar ‘Tocai friulano’ provides compartmentation of grapevine *Flavescence dorée* phytoplasma

**DOI:** 10.1186/s12870-023-04122-0

**Published:** 2023-03-25

**Authors:** Sofia Casarin, Simone Vincenzi, Antonella Esposito, Luisa Filippin, Vally Forte, Elisa Angelini, Nadia Bertazzon

**Affiliations:** 1 Research Centre for Viticulture and Enology (CREA), Via XXVIII Aprile 26, 31015 Conegliano, TV Italy; 2 Department of Agriculture, Food, Environmental and Animal Sciences, University of Udine, Via delle Scienze, 206, 33100 Udine, UD Italy; 3 Department of Agronomy, Food, Natural resources, Animal and Environment (DAFNAE), University of Padua, Viale dell’Università, 16, 35020 Legnaro, PD Italy

**Keywords:** Gene expression, Grapevine yellows, Recovery, Stilbenoids, Symptoms, *Vitis vinifera*

## Abstract

**Background:**

*Flavescence dorée* (FD) is a grapevine disease caused by phytoplasma and it is one of the most destructive pathologies in Europe. Nowadays, the only strategies used to control the epidemics are insecticides against vector, but more sustainable techniques are required. Completely resistant *Vitis vinifera* varieties have not been uncovered yet, but differences in susceptibility among cultivars and spontaneous recovery from FD symptoms have been observed. The grapevine cultivar ‘Tocai friulano’ shows very low susceptibility to FD but its defence strategy to counteract the phytoplasma spread has not been deciphered yet. In this work, the mechanisms occurring within ‘Tocai friulano’ FD-infected plants were examined in depth to identify the phytoplasma distribution and the defence pathways involved.

**Results:**

In ‘Tocai friulano’ symptoms of FD-infection remained confined near the area where they appeared during all the vegetative season. Analyses of secondary phloem showed a total absence of FD phytoplasma (FDp) in the trunk and its disappearance in 2-year-old arms from July to November, which was different from ‘Pinot gris’, a highly susceptible variety.

Diverse modulations of defence genes and accumulation of metabolites were revealed in 1-year-old canes of ‘Tocai friulano’ FD-infected plants, depending on the sanitary status. Symptomatic portions showed high activation of both jasmonate- and salicylate-mediated responses, together with a great accumulation of resveratrol. Whereas activation of jasmonate-mediated response and high content of ε-viniferin were identified in asymptomatic 1-year-old cane portions close to the symptomatic ones.

**Conclusion:**

Successful defence mechanisms activated near the symptomatic areas allowed the compartmentation of FD symptoms and phytoplasmas within the infected ‘Tocai friulano’ plants. These results could suggest specific agronomical practices to be adopted during FD management of this variety, and drive research of resistance genes against FD.

**Supplementary Information:**

The online version contains supplementary material available at 10.1186/s12870-023-04122-0.

## Background

Grapevine yellows (GY), serious diseases of grapevine, spread all over the grape growing regions in the world. They are caused by phytoplasmas, primitive gram-positive bacteria without cell wall living in the phloem and sieve cells of plants and in the haemolymph of insect vectors. In Europe the main GY are *Flavescence dorée* (FD) and *Bois noir* (BN), associated with *Flavescence dorée* phytoplasma (FDp) and ‘*Candidatus* Phytoplasma solani’, respectively. FD is the most destroying GY, a quarantine disease present in Europe since 1950, that caused severe damages and production decline in many European countries [1]. Phytoplasmas associated to FD disease are classified in the phylogenetic 16SrV ribosomal group, subgroups C and D [2], and are transmitted by *Scaphoideus titanus* Ball, a leafhopper of American origin [3]. *S. titanus* lives and feeds on grapevine, transmitting FDp very efficiently from a vine to another. FD symptoms appear on leaves, bunches and canes, generally one year after the inoculation [4]. Leaves of infected grapevines become red in the red varieties and yellow in the white varieties, especially in the veins, and are generally crispy, brittle and downwards rolling. Flowers and/or bunches wither and fall. Canes show short internodes and do not lignify in autumn. A general decline of the plants occurs, year after year. However, plants only partially infected can have good production at the harvest and can survive for many years [5].

Grapevine varieties show differences in susceptibility to FD when growing in the same environmental conditions and exposed to the same disease pressure. Symptom expression, phytoplasma concentration and occurrence of infected plants in the field or in experimental controlled conditions vary according to the *Vitis vinifera* variety [4, 6–9]. Indeed, some varieties, such as ‘Chardonnay’ and ‘Pinot gris’, show very serious damage caused by GY, while others, such as ‘Tocai friulano’or ‘Moscato bianco’, usually have only a few plants that display symptoms, at most in one or two branches. It is known that ‘Tocai friulano’, which is an autochthonous cultivar of the Collio wine-producing area in the Friuli Venezia Giulia region (Northeastern Italy), exhibits very low susceptibly to FD, even though when subjected to high disease pressure. Moreover, symptoms on infected plants of ‘Tocai friulano’ have been frequently observed to disappear from one year to the next [10, 11].

Grapevines infected by FD can show spontaneous and stable remission of symptoms in the following vegetative year, accompanied by the disappearance of the pathogen from the canopy and the restoration of the grape productivity [4, 12]. The recovery phenomenon, associated or not to the absence of phytoplasmas from the host, has been reported in fruit trees as apple and apricot [13, 14] and also in grapevines infected by BN [15]. Factors triggering the recovery process are not well known. External stimuli, such as abiotic stresses, treatments with resistance inducers and antimicrobial molecules have shown to be effective in stimulating recovery from phytoplasmas on different plant species [16]. Space-time analysis of FD epidemics indicated that the course of the infection in vineyard seems to be driven by defence mechanisms induced by the infected plants themselves [17]. Over the last years, healthy, recovered and FD- / BN-infected grapevines were compared using different approaches to identify the molecular markers involved in recovery. Global transcription and protein profiles revealed an altered expression of several genes and proteins in recovered vines compared to the healthy ones, mainly involved in the reprogramming of stress-mediated responses previously triggered by the phytoplasma [7, 18–23]. Moreover, foliar tissue of recovered plants accumulated an increased level of endogenous H_2_O_2_, and higher concentration of specific categories of stilbenoids, such as viniferin [12, 19, 22]. Such studies were performed on stably recovered plants, which are identifiable only after two years of absence of phytoplasma and symptoms, when the remission process is reached.

The possibility of recovery changes significantly according to the grapevine cultivar too [8, 17]. There is no information on the rate of recovery in ‘Tocai friulano’, probably because very few FD-infected plants per vineyard are usually found. Preliminary field observations on ‘Tocai friulano’, carried out over several years of GY monitoring in different vineyards, suggested the ability of this variety to limit the systemic spread of symptoms through the plant. Therefore, it is conceivable that in ‘Tocai friulano’, together with the symptoms, the phytoplasma could also be confined in a part of the plant, which could be in turn eliminated during the usual pruning operation, and that some defence mechanisms are activated in specific portions within a FD-infected plant.

In diseased grapevines, phytoplasmas reside almost exclusively in the phloem sieve tube elements, through which they spread systematically throughout the plant, the flow being guided by the sink-source relation [24]. During dormancy, phytoplasmas persist in the canes and in the permanent structures of the vine, colonizing the plant from one year to the next, even if they have been detected only occasionally, at low titre, in the secondary phloem of canes and in the roots [25, 26]. Few studies, focussing on shoots and canes, demonstrated that other plant structures, besides leaves, could be the site of defence reactions that could lead to the compartmentation of phytoplasmas. Rusjan et al. [27] demonstrated that the lack of lignification observed in shoots/canes of ‘Chardonnay’ BN-infected plants is caused by the defence reaction carried out by the plant itself through the accumulation of many phenolic substances, such as flavonols, flavanols and stilbenoids, involved in resistance to pathogen attack. Moreover, Jelmini and colleagues [28], describing the anatomical modification caused by FDp on stem tissues of the scarcely susceptible cultivar ‘Merlot’, proposed a defence strategy based on the compartmentation of the infected shoots, by preventing the maturation of the vessels and by forming occlusions through tyloses. Several studies that have been performed so far to investigate the effects of phytoplasma infection in grapevine, or to explore the recovery phenomenon, have been carried out only in the leaves, thus evaluating the plants during the vegetative period.

The aim of the present study was to investigate the responses elicited by FDp in canes of ‘Tocai friulano’ grapevines during the growing season and also the dormancy, focussing on molecular and metabolic changes that could be responsible for the containment of the disease. Presuming that the strategy implemented by ‘Tocai friulano’ serves to achieve the recovery status from FD, the research was focussed on some of the mechanisms that have been reported to be active in stably recovered plants. Such responses could be associated to compartmentation of the pathogen in the plant.

## Results

### Symptoms of GY spread within the canopy in plants of ‘Pinot gris’, whereas they remain localized in plants of ‘Tocai friulano’

**Fig. 1:**
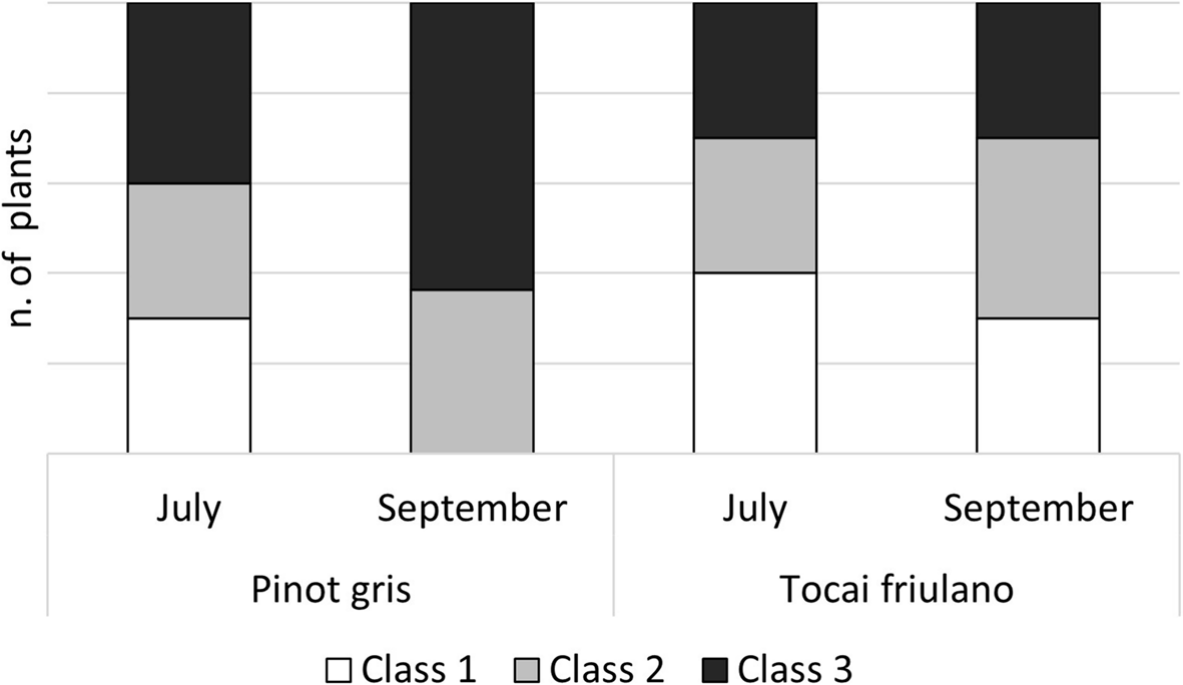
Distribution of plants with different severity of *Flavescence dorée* symptoms in ‘Pinot gris’ and ‘Tocai friulano’. Ten plants per variety were surveyed in July and September of the first year of appearance of symptoms. Three classes of disease were considered: (Class 1) symptoms limited to a small part of one 2-year-old arm (blank); (Class 2) symptoms in most of one 2-year-old arm (grey); (Class 3) both 2-year-old arms with *Flavescence dorée* symptoms (black)

In the surveyed vineyard, the high number of FD-infected plants in the susceptible variety ‘Pinot gris’ (5% in 2019), joined with a high presence of the vector *S. titanus*, led to a natural increase of symptomatic plants in the five neighbouring rows of ‘Tocai friulano’. Indeed, the number of ‘Tocai friulano’ plants displaying the symptoms increased from zero (out of 1380) in 2019 to 6 in 2020 and then 74 in 2021. In the first year of symptom manifestation (2020) ‘Pinot gris’ showed very serious damage associated to GY, while ‘Tocai friulano’ displayed mild symptoms, usually in just one or two branches. In-depth observations of symptoms naturally spreading in the vineyard were conducted in 2021 during the vegetative season. Ten plants of each variety, which were asymptomatic in 2020 and symptomatic in 2021, were identified. All plants were infected by FDp and tested negative for BNp. Characterization of phytoplasmas in the 16S ribosomal region showed that all of them belonged to the 16SrV-D phylogenetic subgroup. In summer 2021 (July), when typical symptoms of FD became clearer, the severity of the disease was very similar between the two varieties, as highlighted by a comparable distribution of plants among the different classes of symptoms (Fig. 1). Towards the end of the vegetative season (September), a difference between the two grapevine varieties appeared, as expected. Indeed, in ‘Pinot gris’ most of the plants were severely affected by the disease: the yellowing and downward curling of leaves and the lack of lignification spread within the canopy during the vegetative season, also causing a strong reduction of grape production. On the opposite, in all the ten plants of ‘Tocai friulano’, symptoms remained enclosed near the area where they appeared, never spreading from one fruiting arm to another, and with slight impairment of grape production. Interestingly, the manifestation of symptoms was often restricted to the apical and median portions of canes, while basal segments showed good lignification and asymptomatic leaves.

### FD phytoplasma is highly occurring in 2-year-old arms and trunk in plants of ‘Pinot gris’, but not in ‘Tocai friulano’

Five plants of each variety, with symptoms restricted to one fruiting arm, were chosen to check the distribution of FDp through different parts of the symptomatic 2-year-old arm (proximal, median, distal) and trunk in summer (July) and during dormancy (November). In general, a higher number of PCR-positive samples was obtained from ‘Pinot gris’ plants in comparison to ‘Tocai friulano’ (83% *versus* 25%, respectively) (Additional file 1). Considering July and November samplings separately, ‘Pinot gris’ showed a similar number of positive samples between the two collection times, even if some portions of the arms had undergone drying out. Conversely, FDp was not found in any of the samples collected from ‘Tocai friulano’ in November. Interestingly, the analyses revealed the absence of the pathogen in the trunk of ‘Tocai friulano’ grapevines, both in July and November, differently from ‘Pinot gris’, where its presence was detected in most of the samples collected from the trunks.

**Fig. 2:**
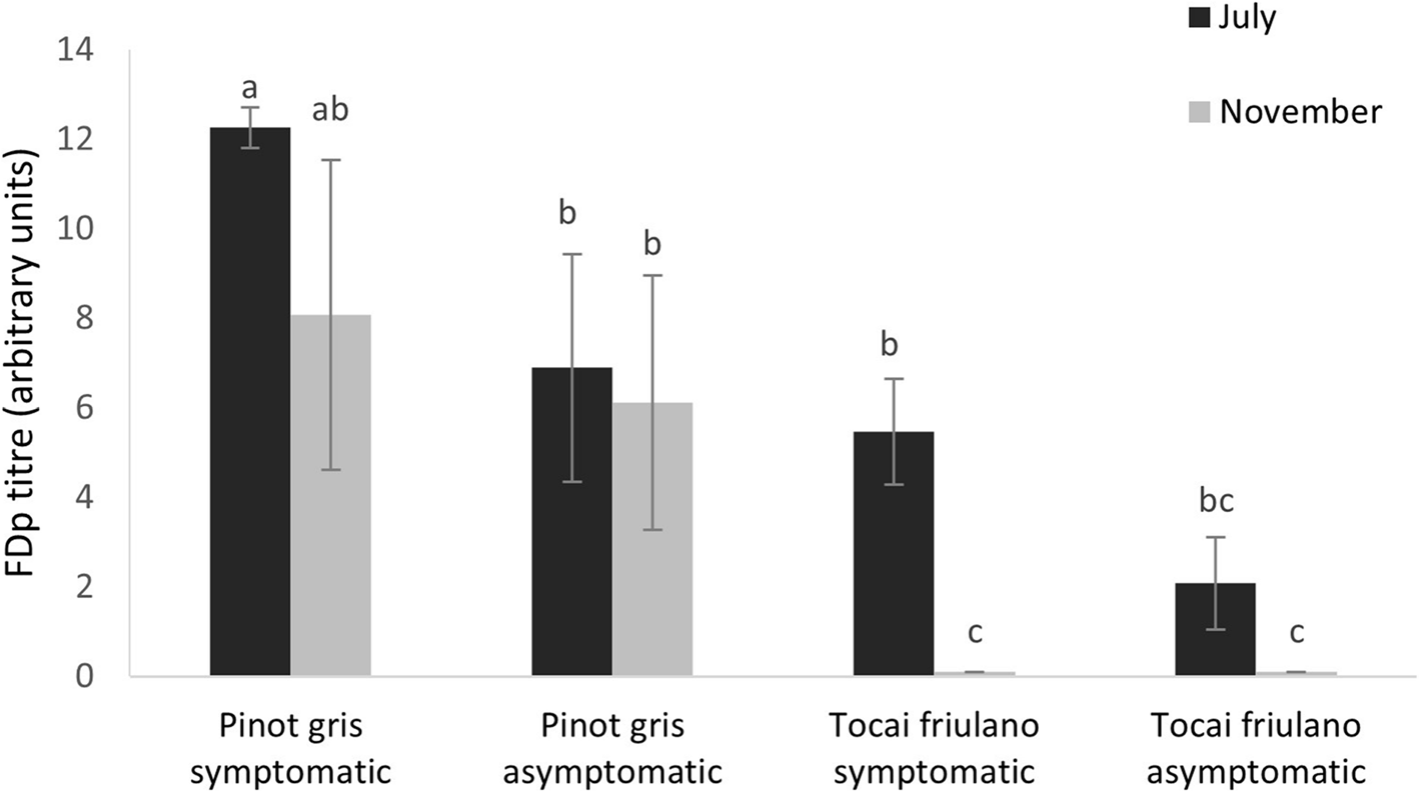
Titre of Flavescence dorée phytoplasma in 2-year-old arms of ‘Tocai friulano’ and ‘Pinot gris’. Relative quantification of the *Flavescence dorée* phytoplasma (FDp), expressed as arbitrary units (log2), in different symptomatic and asymptomatic parts of the 2-year-old arms in ‘Pinot gris’ and ‘Tocai friulano’ infected grapevines. Samples were collected in July, when typical symptoms of *Flavescence dorée* became clear (dark bars), and in November, during dormancy (grey bars). Each value represents the mean ± SE of a variable number of biological replicates (from 5 to 10). Significant differences are indicated by different letters, as determined by the Student-Newman-Keuls test (*P* ≤ 0,05)

The relative quantification of FDp was evaluated comparing proximal, median and distal 2-year-old arm portions from which symptomatic canes grown with those from which asymptomatic canes grown (Fig. 2). In ‘Pinot gris’, a significant higher FDp titre was observed in symptomatic samples compared with the asymptomatic ones in July, but not in November. In ‘Tocai friulano’ the FDp was detected only in samples collected in July, with a titre tendentially lower in asymptomatic samples compared to symptomatic ones. Samples collected from symptomatic 2-year-old arm portions in July harboured a significantly higher FDp titre in ‘Pinot gris’ compared to ‘Tocai friulano’. In November, FDp was detected only in symptomatic and asymptomatic portions of ‘Pinot gris’, and it was never found in ‘Tocai friulano’.

### Focus on ‘Tocai friulano’: different mechanisms occur near symptomatic portions of 1-year-old canes within an infected plant

Further investigations were focussed on ‘Tocai friulano’ to confirm the localization of both symptoms and FDp to a few organs of the infected plants during one vegetative season. Canes and leaves, with or without symptoms, were sampled from selected plants in September 2021 and then tested for the presence of FDp. Real-time RT-PCR identified FDp in veins of yellow and down-rolled leaves and in the phloem collected from the corresponding segment of non-lignified canes (S, see material and methods). All the tested basal portions of partially symptomatic canes (AS), showing good lignification, were negative for the FDp as well as the respective asymptomatic leaves (Table 1). Likewise, all the asymptomatic canes and leaves, grown on symptomatic (AA) or asymptomatic (A) 2-year-old arms of FD-infected plants, were found FDp-free, such as asymptomatic canes from asymptomatic plants (H).

**Table 1:**
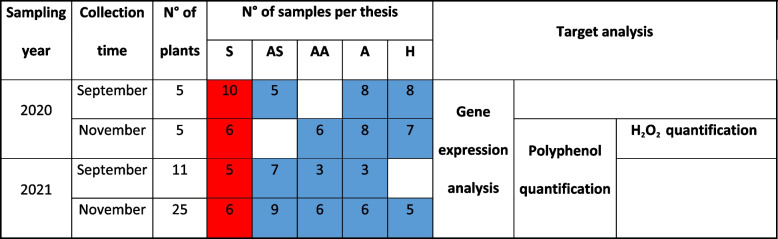
Number of samples collected from 1-year-old canes of Tocai friulano plants

Total number of secondary phloem samples for each thesis (S, AS, AA, A, H) collected from 1-year-old canes of Tocai friulano plants. A variable number of healthy and *Flavescence dorée*-infected plants of the cultivar Tocai friulano were sampled for two years (2020 and 2021), in September and November, corresponding to the end of the vegetative season and the dormant phase, respectively. Timing and total number of samples for each kind of analysis (gene expression, evaluation of polyphenol and H_2_O_2_ contents) are indicated. Positive (red) and negative (blue) results of molecular analysis for the presence of FDp, performed both on leaves and canes, are indicated.

Different approaches were used to deepen the molecular processes underlying the localization of both symptoms and FDp infection to a few parts of the plant, focussing on the basal portions of 1-year-old canes. Changes in transcription of some categories of genes were investigated in 2020 and 2021 on a panel of 30 genes (Additional file 2). The transcript levels obtained from the different theses (S, AS, AA, A and H) were compared: most of the genes analysed in 2020 showed similar transcript abundance between A and H canes, thus H samples were disregarded in 2021. Moreover, accumulation of stilbenes and polyphenols, and the H_2_O_2_ content were measured in some of the samples collected for gene expression study.

#### Most genes involved in recovery of grapevine from FD are up-regulated only in S samples, with the exception of some stilbene synthases

Several genes, known to be modulated during the recovery of grapevine from FD or BN diseases, were initially selected, although literature data were obtained in leaves, whereas in the present paper the focus was on canes.

The analysis was first addressed to investigate the transcript changes of some genes involved in production and scavenging of H_2_O_2,_ chosen from the works of Gambino et al. [19] and Prezelj et al. [29] (Additional file 2). Three genes encoding for a glycolate oxidase (GOX) and two germin-like proteins (GLP1 and GLP3), implicated in H_2_O_2_ accumulation, were up-regulated in S samples in 2020 and 2021, especially in November, while no differences were observed among AA or AS canes compared to A and H samples, in the two sampling periods, except for *GLP1* which was up-regulated in AS samples in September 2021. Similarly, three genes coding for scavenging enzymes, i.e. catalase (*CAT1*), glutathione peroxidase (*GPX4*) and ascorbate peroxidase (*APX3*), showed higher expression level in S samples compared to the other theses. On the opposite, *ascorbate peroxidase 6* (*APX6*) showed lower transcript level in S than AA, A and H in November 2020.

Another two genes, a member of the WRKY transcription factor family (*WRKY2*) and a GDSL esterase/lipase (*GDSL1*), were studied (Additional file 2). In the woody plant material studied here, for all the collection timing, no significant difference was detected in transcript abundances of both genes in the asymptomatic basal portions of AS and AA canes compared to A thesis, in contrast, they were up-regulated in S samples.

Analysis of a *callose synthase* gene (*CAS)*, involved in callose deposition, revealed a higher transcript level in S samples only in November samplings, while no differences were observed among samples collected in September (Additional file 2). A peroxidase involved in the reinforcement of the cell wall (*PER*) showed the same results as *CAS* gene; in addition, a higher expression level in AS samples compared to AA and A samples was observed in November 2021 (Additional file 2).

**Fig. 3:**
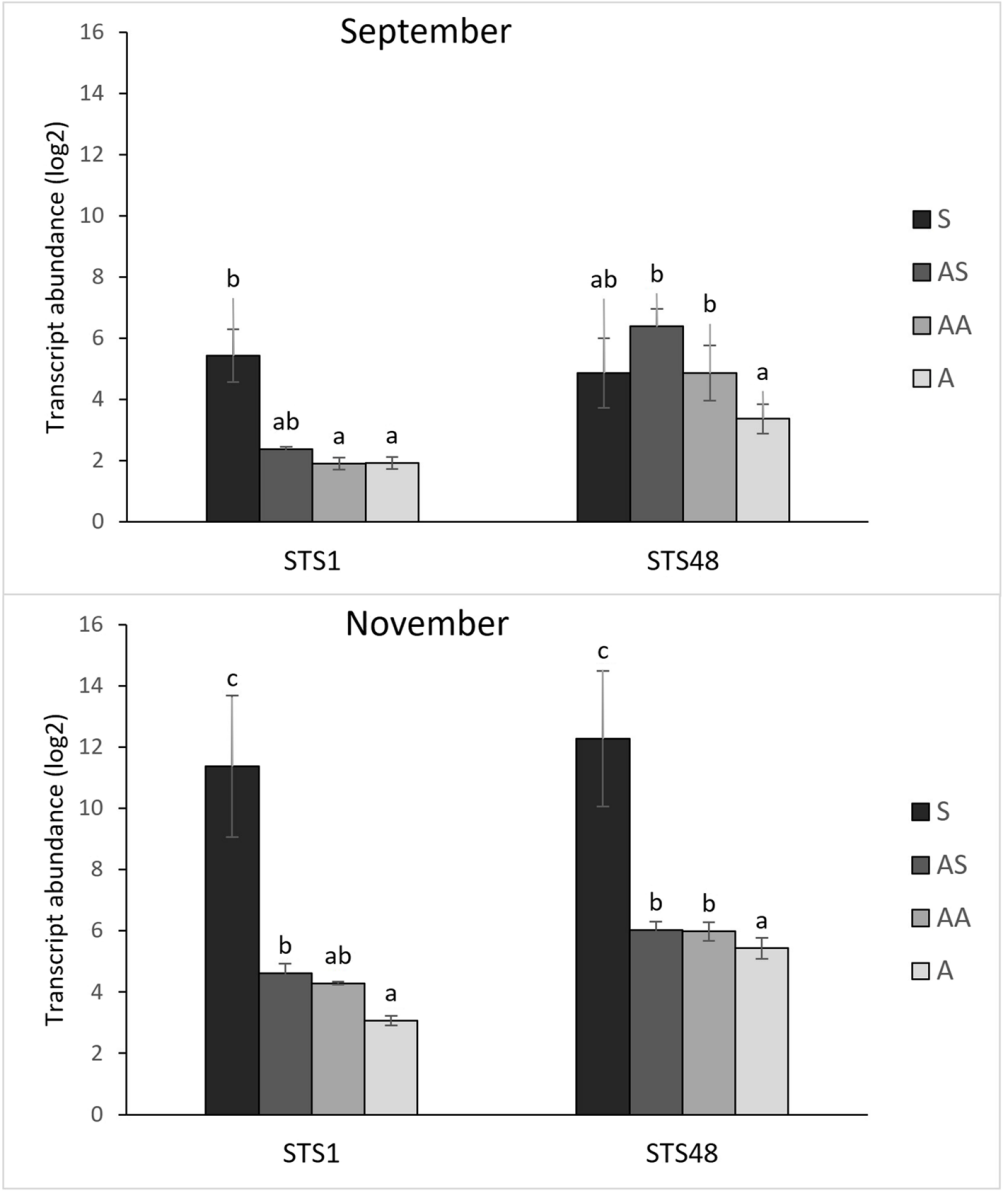
Relative expression levels of two** stilbene synthase **genes. Relative quantification, expressed as arbitrary units (log2), of *STS1* and *STS48* transcripts obtained from 1-year-old canes grown from the symptomatic (S, AS and AA) and asymptomatic (A) 2-year-old arms of *Flavescence dorée* infected ‘Tocai friulano’ plants collected in September and November 2021. Normalisation of transcriptional levels was performed using *GAPDH* and *COX* genes as endogenous controls. Significant differences (*P* ≤ 0.05) among treatments are indicated by different letters, according to parametric one-way ANOVA using Student-Newman-Keuls test

Three genes coding for stilbene synthase (*STS1*, *STS16* and *STS48*), involved in the phenolic pathway, have been investigated (Additional file 2). In 2020, *STS* genes showed to be all significantly up-regulated in S samples compared to A and H theses. To a lesser extent, *STS1* and *STS48* were also up-regulated in AA and in both AA and AS samples, compared to H canes, respectively (Additional file 2). Analysis carried out in 2021, on a larger number of samples, confirmed higher transcript level of *STS1* and *STS16* in S samples compared to other theses. Moreover,, a higher transcript level of *STS1* was observed in AS samples collected in November, while *STS48* was significantly up-regulated in both AA and AS canes in the two samplings compared to the asymptomatic canes A (Fig. 3).

#### Genes of the salycilate-mediated defence response are up-regulated only in S samples, while some genes of the jasmonate-mediated pathways are up-regulated in S, AS and AA samples

The activation of different sets of defence-related genes is mediated by an interconnected network of signal transduction pathways depending mainly on hormones, such as salicylic acid (SA), jasmonic acid (JA) and ethylene (Et).

In the present work the activation of the salycilate-mediated defence response in canes was investigated by monitoring the expression levels of three downstream SA-responsive genes, encoding for the pathogenesis-related proteins PR1, PR2 and PR5, and of a gene involved in the SA-signalling, namely *nonexpressor of pathogenesis-related genes 1* (*NPR1*) (Additional file 2). The genes encoding for PR1, PR2 and PR5 were strongly up-regulated in S samples in all the collection time points compared to AS, AA and A samples, which showed no differences among them; moreover, an up-regulation of *NPR1* was observed in S canes collected in September 2021 and in both years in November. Additionally, a member of the WRKY transcription factor family (*WRKY70*), known as an activator of SA-induced genes [30], was consistently up-regulated only in S samples, with the only exception of September 2020, when the AS samples were also up-regulated compared to A and H canes (Additional file 2, Fig. 4). In the present work, for all the sampling periods, the expression level of *DMR6*, a gene involved in the SA homeostasis, was significantly higher in S samples than in asymptomatic ones (A). Interestingly, the transcription of the same gene was always up-regulated in AS samples compared to A canes, and also in the AA canes, but only for the sampling performed in November 2021 (Additional file 2, Fig. 5).

**Fig. 4:**
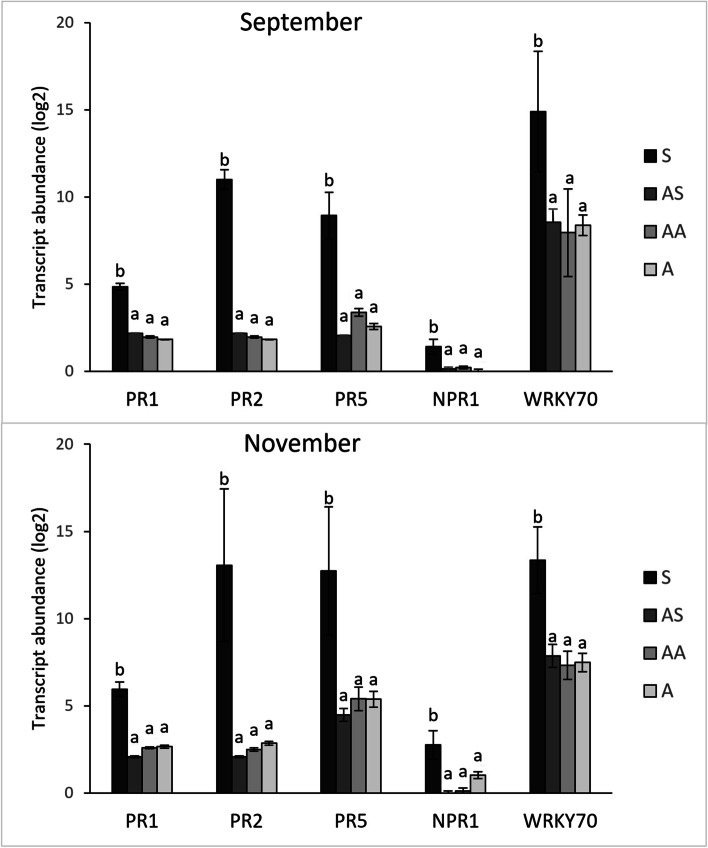
Relative expression levels of genes involved in salicylate-mediated response. Relative quantification, expressed as arbitrary units (log2), of selected genes involved in salycilate-mediated defence response (*PR1, PR2, PR5, NPR1* and *WRKY70*) obtained from 1-year-old canes grown from the symptomatic (S, AS and AA) and asymptomatic (A) 2-year-old arms of *Flavescence dorée* infected ‘Tocai friulano’ plants collected in September and November 2021. Normalisation of transcriptional levels was performed using *GAPDH* and *COX* genes as endogenous controls. Significant differences (*P* ≤ 0.05) among treatments are indicated by different letters, according to parametric one-way ANOVA using Student-Newman-Keuls test

**Fig. 5:**
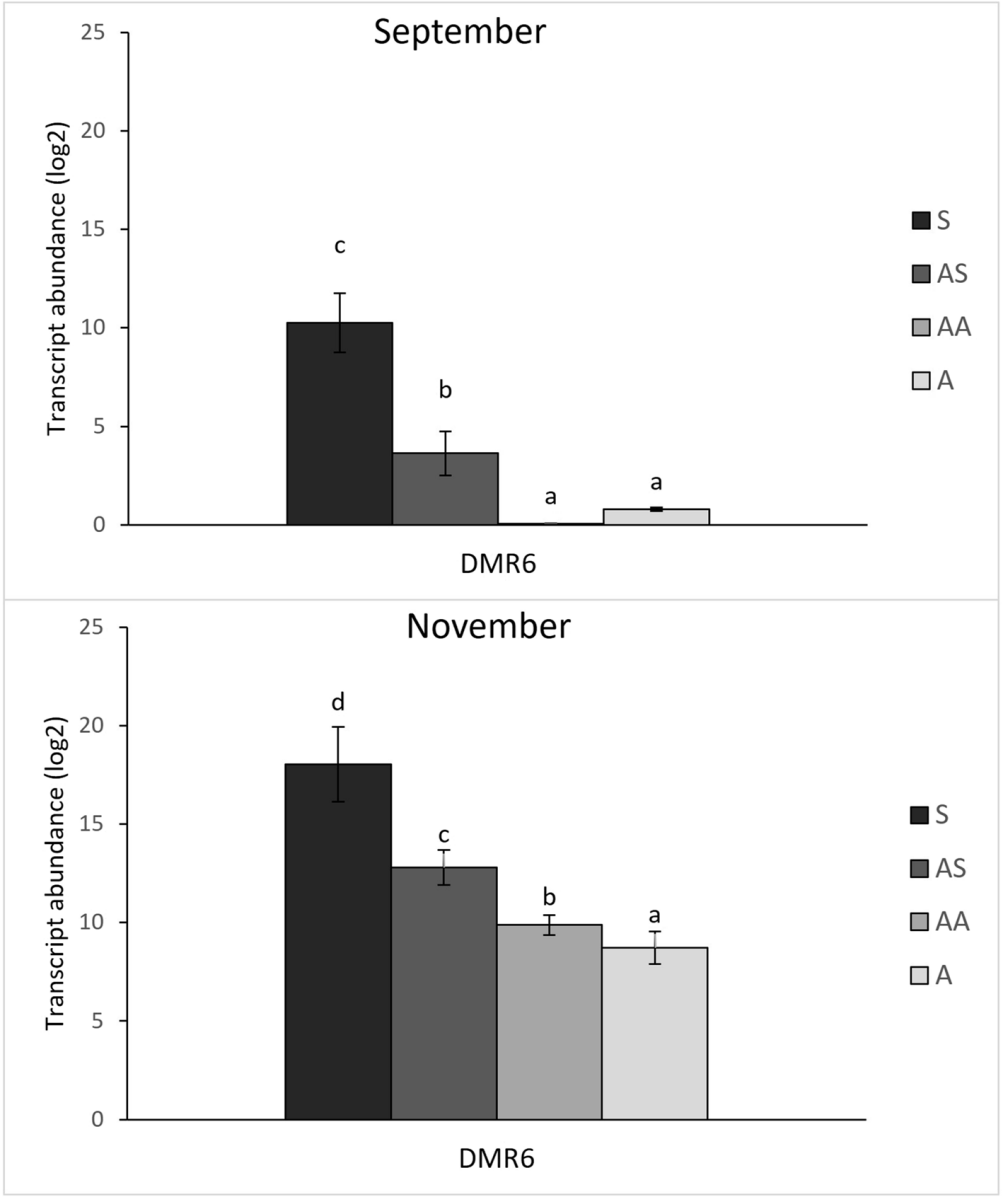
Relative expression levels of** DMR6 **gene. Relative quantification, expressed as arbitrary units (log2), of *DMR6* gene obtained from 1-year-old canes grown from the symptomatic (S, AS and AA) and asymptomatic (A) 2-year-old arms of *Flavescence dorée* infected ‘Tocai friulano’ plants collected in September and November 2021. Normalisation of transcriptional levels was performed using *GAPDH* and *COX* genes as endogenous controls. Significant differences (*P* ≤ 0.05) among treatments are indicated by different letters, according to parametric one-way ANOVA using Student-Newman-Keuls test

**Fig. 6:**
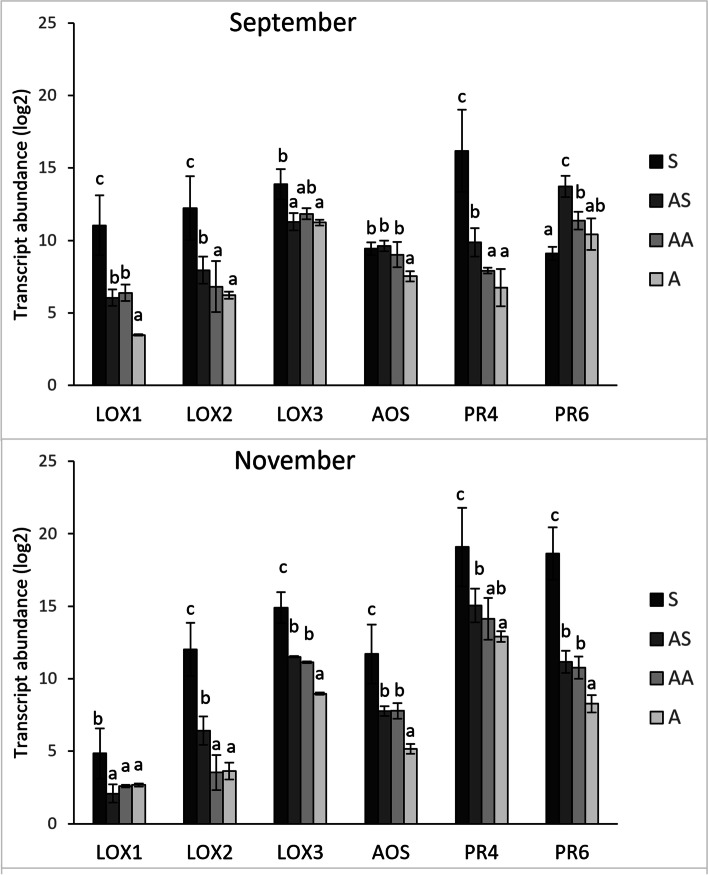
Relative expression levels of genes involved in jasmonate-mediated response. Relative quantification, expressed as arbitrary units (log2), of some genes involved in jasmonate-mediated defence response (genes encoding lipoxygenases *LOX1, LOX2, LOX3, allene oxide synthase AOS, PR4* and *PR6*) obtained from 1-year-old canes grown from the symptomatic (S, AS and AA) and asymptomatic (A) 2-year-old arms of *Flavescence dorée* infected ‘Tocai friulano’ plants collected in September and November 2021. Normalisation of transcriptional levels was performed using *GAPDH* and *COX* genes as endogenous controls. Significant differences (*P* ≤ 0.05) among treatments are indicated by different letters, according to parametric one-way ANOVA using Student-Newman-Keuls test

To examinate the JA-mediated defence response in canes, three genes encoding 13-lipoxygenases (named *LOX1, LOX2* and *LOX3*), one *allene oxide synthase* (*AOS*) and one *jasmonate resistant 1* (*JAR*), were selected from the JA biosynthetic pathway. In addition, the expression of three genes coding for jasmonate-responsive PR proteins, *PR4*, *PR6, PR10* and *PR12,* was investigated (Additional file 2). As observed for the SA-responsive genes, the genes involved in the JA-mediated-defence response showed higher transcript level in S samples in most of the sampling dates. Apart from *JAR*, *PR10* and *PR12*, which did not show transcriptional changes between AS, AA and A samples, interesting results were obtained for the other genes. Analysis performed in 2020 revealed that *LOX1, LOX3, PR4* and *PR6* genes were up-regulated in AS compared to A samples collected in September and expression level of *LOX1, AOS* and *PR4* genes was higher in AA than A samples collected in November (Additional file 2). Expression study carried out in 2021 confirmed that most of JA-mediated defence response genes were up-regulated in AS and/or AA samples in comparison to A samples (Fig. 6).

Lastly, *ACS*, a key-gene of the Et-mediated response, was included in the present study (Additional file 2). Its expression profile showed no variation in samples collected in September; conversely, a higher transcript level was detected in S samples, compared to the others, in November.

#### ε-viniferin accumulates mainly in AA samples, while S samples show higher amount of resveratrol

Alteration of the phenylpropanoid pathway, leading to synthesis of flavanone, flavanols, flavonols and stilbenoids, has been studied.

Polyphenol analysis of ‘Tocai friulano’ FD-infected plants, sampled in September (2021) and November (2020, 2021), was performed to evaluate the content of stilbenoids, such as resveratrol and ε-viniferin, and proanthocyanidins B2, catechin and epicatechin in canes.

**Fig. 7:**
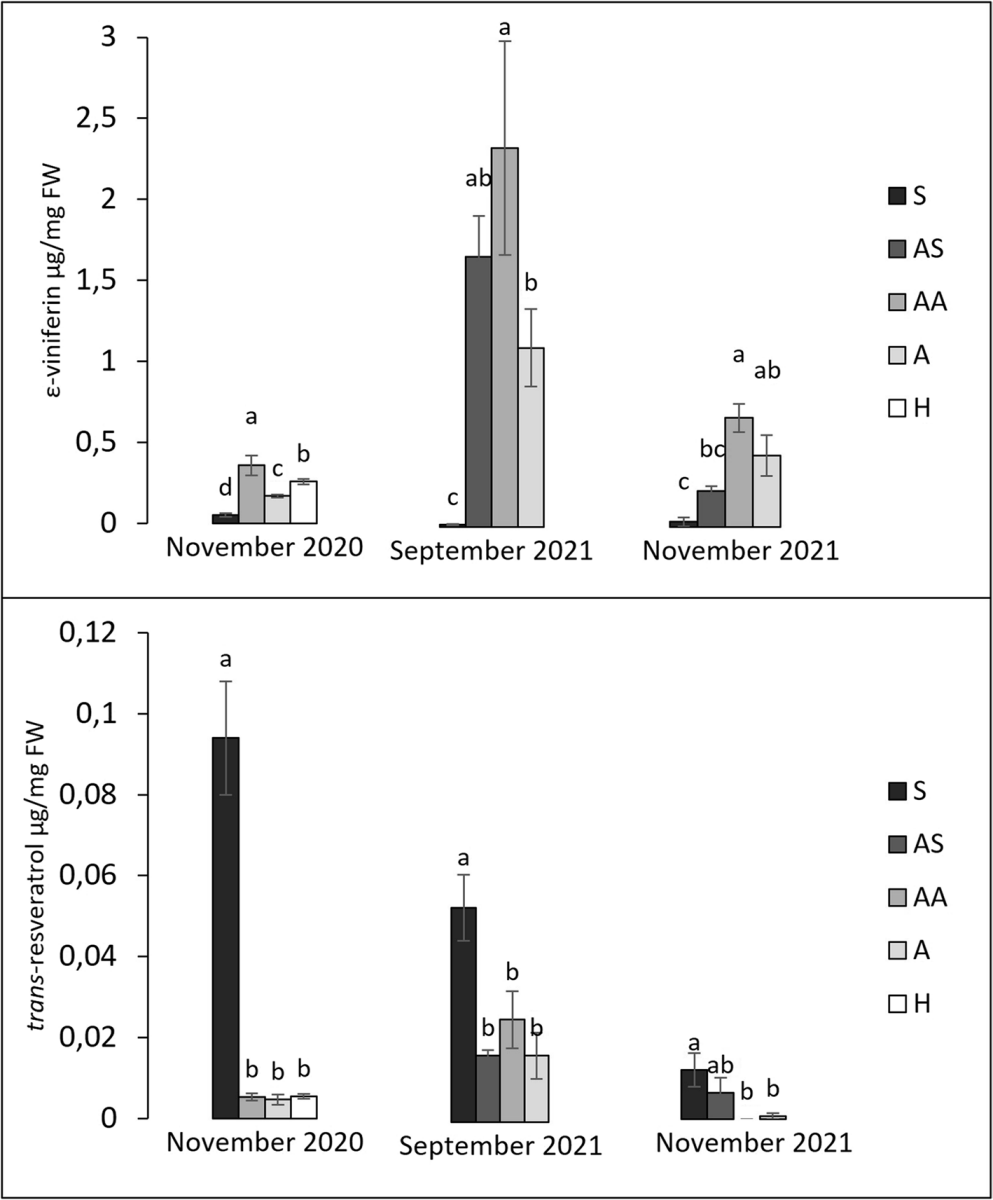
Stilbenoids content: A) *trans*-resveratrol; B) ε-viniferin. Samples were collected from 1-year-old canes grown from the symptomatic (S, AS and AA) and asymptomatic (A) 2-year-old arms of *Flavescence dorée* infected ‘Tocai friulano’ plants in September (2021) and November (2020 and 2021), and from healthy plants (H) in November (2020). Significant differences (*P* ≤ 0.05) among treatments are indicated by different letters, according to parametric one-way ANOVA using Student-Newman-Keuls test

In the present study, higher levels of resveratrol were obtained in S samples compared to the others, especially in November 2020 and September 2021, when, on the opposite, the highest contents of ε-viniferin were detected in AA samples. The content in ε-viniferin decreased from September to November for AS, AA and A samples, while values measured in S samples remained very low for every time point (Fig. 7).

Proanthocyanidins, also known as condensed tannins, are a group of flavonoids derived from flavan-3-ols (catechins and epicatechins. S samples accumulated significant higher amounts of proanthocyanidins B2 in all the collection time points, and catechins only in November 2021. On the opposite, only few differences were obtained for epicatechins in November 2020 (Additional file 3).

#### S samples accumulate less H_2_O_2_

Analysis of the content in H_2_O_2_ was performed in November 2020 on samples collected from 1-year-old canes belonging to the thesis S, AA, A and H, and the lowest amount was measured in S samples. No significant difference was observed between AA, A and H samples (Additional file 4).

## Discussion

Differences in susceptibility to FD between *V. vinifera* cultivars have been observed in vineyards and under controlled conditions based on disease prevalence, symptom severity and plant’s ability to recover [6, 8, 9]. Cultivars like ‘Chardonnay’ and ‘Pinot gris’ can face serious FD epidemics and significant production losses. On the opposite, evidence for very low susceptibility to FD has been reported for the cultivar ‘Tocai friulano’ [10, 11]. In the present paper the behaviour of ‘Pinot gris’ and ‘Tocai friulano’ FD-infected plants was compared starting from the first appearance of symptoms. Differences in susceptibility to FD have been observed already during one vegetative season. Indeed, in summer (July), when the typical symptoms of FD became evident, ‘Tocai friulano’ showed very similar disease severity compared to ‘Pinot gris’, while at the end of the vegetative season (September) symptoms in ‘Tocai friulano’ remained confined near the area where they appeared, whereas the symptoms were widespread in ‘Pinot gris’ canopy. A different behaviour has been reported by Roggia et al. [9] in the comparison between ‘Barbera’ and ‘Nebbiolo’ cultivars, showing high and low FD-susceptibility, respectively. In that case, more severe symptoms had been observed during spring and early summer in infected vines of ‘Barbera’ than ‘Nebbiolo’, while, on the opposite, the differences disappeared in late summer, as symptoms worsened in both varieties. This suggests that the low susceptibility to FD typical of ‘Tocai friulano’ and Nebbiolo could involve different defence strategies. In the case of ‘Tocai friulano’, observations carried out in this paper suggest that the compartmentation of symptoms and phytoplasmas plays a key role in its response to FD.

The presence of BN/FD symptoms has been associated with the occurrence of phytoplasmas in leaf tissue [4, 18]. In this work, it was shown that this correlation can be also applied to 1-year-old canes in ‘Tocai friulano’, as FDp was detected only in non-lignified symptomatic cane portions. Differently, the analyses performed on the partially symptomatic 2-year-old arms in both varieties allowed FDp detection, depending on the season and the variety, even in the portions from which asymptomatic canes grown (Fig. 2). After the onset of symptoms on some leaves and 1-year-old canes, the translocation of FDp to other parts of the canopy involves the passage through the secondary phloem of older structures, such as arms and trunk. In the present paper, in summer, when the manifestation of the disease was very similar between ‘Tocai friulano’ and ‘Pinot gris’, FDp was detected in the symptomatic 2-year-old canes of both varieties, while, after the end of the vegetative season, it was found only in the arms of ‘Pinot gris’. Thus, the diagnosis of FDp in the secondary phloem of the fruiting arms of FD-infected grapevines revealed a close correlation between the presence of FDp in these parts of the plants and the distribution of the symptoms over time in the two cultivars. Higher FDp titres were always measured in ‘Pinot gris’ than in ‘Tocai friulano’; in ‘Pinot gris’ the asymptomatic parts of 2-year-old arms, whatever the sampling period, harboured similar or even slightly higher FDp titres than the symptomatic portions of ‘Tocai friulano’ plants. Moreover, results obtained from the analyses on the trunk sections correlated with the widespread distribution of FD symptoms occurring in ‘Pinot gris’ grapevines in late summer and with the delimitation of symptoms in ‘Tocai friulano’ plants during the growing season. Indeed, FDp was detected in most of the samples collected from trunks of ‘Pinot gris’ plants, both in July and November, differently from ‘Tocai friulano’, where it was never detected in the trunk. These results suggest the ability of ‘Tocai friulano’ to enclose FDp in the aerial part of the plants, and particularly in the 1-year-old canes with symptoms, during the vegetative season. This was still more evident in partially symptomatic canes (AS), where FDp passed through the whole new shoot and reached the sprouts or/and leaves in spring when the shoot was growing, remained in the upper part of the shoot till autumn, while it was not detectable anymore in the basal part in autumn. However, it is not possible to completely exclude the presence of a few cells of FDp in these or other parts of the plant, not detectable with the protocol used in the present study, or its presence in plant portions not sampled. Some works have shown that the diagnosis of phytoplasmas in tissues other than leaves allowed the detection of a low proportion of positive samples. In grapevine the phytoplasma detection from cane vascular tissues, cordons and trunks was reported for ‘*Candidatus* Phytoplasma australiense’ (16SrXII-B) and Tomato big bud phytoplasma (16SrII-D), associated with Australian Grapevine Yellows [31], and also for BNp and FDp [25, 32]. In these works, the phytoplasmas were detected in various grapevine tissues throughout the year but were not always detected in all replicates. In general, the explanation of these results was attributed to the uneven distribution of phytoplasmas, or to the seasonal influence on the efficiency of detection, and/or to the low titre of phytoplasma. Unlike the studies reported above, based on phytoplasma DNA detection, in the present work the diagnosis of FDp in the secondary phloem of 1-year-old canes, arms and trunks was carried out with an optimized procedure for the extraction of high-quality RNA from woody tissues, which are rich in polysaccharides and polyphenolic compounds, to avoid inhibition of PCR reaction [33]. The results obtained with this diagnostic method appear reliable for at least three reasons. First, the results obtained from the secondary phloem of 1-year-old canes were confirmed by the results obtained from the correspondent leaves: FDp was detected only in samples collected from symptomatic leaves and from the relative non-lignified 1-year-old canes, while asymptomatic canes tested always negative. Secondly, as reported above, a correlation emerged between the presence of FDp in the arms of ‘Pinot gris’ and ‘Tocai friulano’ and the spread of the symptoms in the tested plants. Third, the more susceptible cultivar ‘Pinot gris’ had high FDp titres on arms and trunks, while in the slightly susceptible ‘Tocai friulano’ the FDp was poorly multiplying in these organs. This result, obtained on woody tissue, agrees with those reported on symptomatic leaves, showing, generally, higher phytoplasma titres harboured on more susceptible *V. vinifera* cultivars [6]. For instance, FDp titre in symptomatic leaves of Nebbiolo, a scarcely susceptible variety, has been reported to be lower than in Barbera, a highly susceptible one [9, 34].

Taken all together, these results suggested that the strategy adopted by ‘Tocai friulano’, but not by ‘Pinot gris’, to avoid the spread of FDp and FD symptoms to the whole canopy and to the permanent tissues of the infected plants, could be related with its less susceptible behaviour against FD disease. Further analyses in the secondary phloem of ‘Tocai friulano’ canes were performed to better understand the transcriptomic and metabolic changes happening in the tissues close to the symptomatic ones, which could play a role in limiting the disease spreading, focussing on some of the defence mechanisms and metabolites that have been reported in stably recovered plants. Analyses were performed in September and November, after the cane lignification process took place and when FD symptoms were evident not only on leaves but also on canes.

On the whole, similar production of polyphenols and H_2_O_2_, as well as comparable gene expression, were detected between ‘Tocai friulano’ canes collected from healthy plants (H) and those grown on asymptomatic arms of FD-infected plants (A), which showed, on the opposite, wide differences with respect to symptomatic canes (S) collected from the same vine. Gene expression analyses revealed the upregulation of almost all the tested genes in the S canes, compared to the asymptomatic ones, especially when sampled during dormancy (Additional file 2). These results demonstrate that dramatical transcriptional changes, widely reported for FD-infected leaves [19, 22, 34], occur in symptomatic woody canes as well. In the present paper, a lower amount of H_2_O_2_, previously reported by Gambino et al. [19] in leaf midribs of FD-infected plants in comparison to healthy ones, has been shown to take place in the secondary phloem of symptomatic canes (Additional file 4), although tested genes involved in H_2_O_2_ production (*GOX*, *GLP1* and *GLP3*) were mainly up-regulated in these tissues at the sampling time points. This up-regulation of most of the genes encoding for scavenging enzymes (*CAT1*, *APX3, GPX4*) may be involved in the lower accumulation of H_2_O_2_ in symptomatic canes. Furthermore, in comparison to A samples, S canes accumulated greater amounts of some antimicrobial compounds (Fig. 7, Additional file 3), such as proanthocyanidins and resveratrol, the latter being in agreement with the upregulation of three *STS* genes (*STS1*, *STS16* and *STS48*) (Fig. 3). A higher level of total stilbenoids was reported by Pagliarani et al. [22] in veins collected from FD-infected plants *versus* healthy ones, although no significant differences was found for resveratrol. Conversely, a study carried out on grapevine canes showed that the concentration of many stilbenoids, such as resveratrol, and many flavanols, such as procyanidins, was significantly increased in the presence of BN infection [27]. As a response to BNp invasion, the synthesis of defence compounds, starting from the same precursors of lignan and lignin, may affect the lignification process of canes [27]. Similarly, the lack of lignification, observed in S canes, may have been caused by the alteration of the phenylpropanoid pathway, which, together with the severe transcriptional modifications mentioned above, could be part of a strong, but generic, defence response of ‘Tocai friulano’ to counteract the FDp. Moreover, other works reported that, in grapevine leaves infected with BNp or FDp, most JA and SA biosynthetic genes showed up-regulation, as compared to uninfected plants [19, 23]. Indeed, in the present paper the expression profiles of most SA- and JA-responsive genes were up-regulated in S canes compared to A/H samples (Figs. 4 and 6), suggesting that full activation of both SA/JA-mediated responses are not effective in inducing resistance against FDp. It is commonly accepted that SA promotes resistance against biotrophic pathogens, whereas JA and Et usually act as defence against necrotrophic ones [35]. The interaction between SA and JA/Et typically acts in antagonistic way, but there is also evidence of synergic interactions [36]. The crosstalk between different hormonal pathways during phytoplasma infection depend on specific pathosystem; however, most of the studies on plant-phytoplasma interactions showed an upregulation of SA-signalling, mainly ineffective in conferring resistance against the disease [37].

Different results were obtained in the 1-year-old canes grown from the symptomatic 2-year-old arms and showing absence of foliar symptoms and good lignification in the base (AS and AA), when compared with S or A canes within an FD-infected plant. Among the genes selected from literature as leaf markers of recovery from FD disease, *STS1* and *STS48* resulted up-regulated in AS samples and AS and AA samples, respectively, compared to A canes, albeit to a lesser extent than in S samples (Fig. 3). Stilbene synthases catalyse the biosynthesis of the stilbene backbone from the intermediates of the general phenylpropanoid pathway, leading to the production of the basic unit of *trans*-resveratrol and its derivatives [38]. Interesting differences emerged in the stilbenoid contents of different samples (Fig. 7). Indeed, greater and lower accumulations of ɛ-viniferin were detected in AA and S samples, respectively, for all time points analysed. In the case of S samples, low levels of ɛ-viniferin always corresponded to high quantities of resveratrol. The latter does not possess a high antimicrobial activity [39], while high toxicity has been reported for viniferin [38]. Pezet et al. [40] showed that, on downy mildew resistant grapevine cultivars, resveratrol synthesized upon *P. viticola* infection was rapidly oxidized into toxic viniferins, while in susceptible grapevine it was glycosylated into the non-toxic piceid. Pagliarani et al. [22] measured higher level of piceid in FD-infected leaves; on the opposite, upregulation of many *STS* genes, above all *STS48*, and accumulation of ɛ-viniferin have been exclusive found in leaves of stably recovered plants from FDp. Therefore, within an FD-infected plant of ‘Tocai friulano’, accumulation of ɛ-viniferin in AA canes, near the symptomatic ones, suggests the creation of an inhospitable environment for FDp, rich in antimicrobial compounds, to stop the systemic infection. In AS and AA samples, the presence of an effective defence response against FDp was consistent with the activation of the JA-responsive pathway, without the SA-signalling (Figs. 4 and 6). Indeed, genes encoding for PR1, PR2, PR5, and WRKY70, known as marker of the SA-mediated defence response, which were significantly up-regulated in S canes, did not show any transcriptional difference between AS, AA and A samples (Fig. 4). On the opposite, many genes involved in biosynthesis and signalling of JA (*LOX* and *AOS*) and two JA-responsive PR genes (*PR4* and *PR6*) were mainly up-regulated in AS or AA samples compared to A canes (Fig. 6). Activation of JA-mediated pathway has been previously reported in leaves of grapevine recovered from FDp and BNp [22, 23]. The JA pathway appears to be crucial in the defence response of grapevine against phytoplasmas and lots of data suggested the importance of decreased JA levels for the success of phytoplasma infection [19, 23, 37, 41]. Activation of the SA-signalling pathway, that is associated with the presence of phytoplasmas, appears to occur more to compete with the effective JA-response in infected plants, rather than to induce resistance against the disease [19, 23, 37, 41]. Moreover, phytoplasmas have been found to produce the SAP11 effector, which targets a family of plant transcription factors able to suppress the JA-regulated defences, thus ensuring the pathogen success in infection [42]. Indeed, in grapevine susceptible varieties, FDp has shown the ability to repress the JA-mediated defence reaction elicited by its insect vector in the early stages of infection [43]. In stably recovered plants, activation of defence genes linked to JA-signalling and suppression of the SA-signalling have been shown to be crucial in the achievement and maintenance of the recovery status [22, 23]. Analogously, in the present paper, within a FD-infected plant of ‘Tocai friulano’, genes associated with JA/SA metabolism and signalling were induced and repressed, respectively, only in the secondary phloem of asymptomatic canes (AS and AA) close to the symptomatic ones. Interestingly, the expression level of *DMR6*, a gene coding for a SA 5-hydroxylase, with a role in SA homeostasis [44], in AS samples was lower than in S canes and higher than in A samples for all collection times. *DMR6* has been suggested as a susceptibility gene in a class of plant immunity suppressors [45]. Indeed, *Arabidopsis* mutant for *DMR6* showed low susceptibility to downy mildew [46]. The lower expression level of *DMR6* in AS and AA canes, compared to S canes, may be associated with a suppression of the SA-mediated response in favour of the JA-dependent one. These transcriptional changes involving hormonal balance may have affected secondary metabolism, leading, for example, to the observed increase in toxic viniferins. Taken all together, the results suggest that, within a ‘Tocai friulano’ plant inoculated with FD, some strategies, similar to those involved in recovery, were implemented in proximity to the symptomatic zone. No accumulation of H_2_O_2_, which has been largely associated with the recovery from phytoplasma-associated diseases, was observed in AS and AA canes in comparison with A samples, and, in most of cases, nor different modulation of genes involved in the production of H_2_O_2_ or coding for scavenger enzymes. However, literature data refer to H_2_O_2_ foliar phloem level measured on grapevines after two years from the spontaneous remission of FD symptoms [12, 19]. It is not known whether, in recovered plants, H_2_O_2_ accumulation occurs in tissues other than leaves or when it begins during the process of reaching the state of recovery.

## Conclusions

This work suggested that the low susceptibility of ‘Tocai friulano’ originated from its ability to avoid the spread of FDp and FD symptoms to the whole canopy and to the permanent tissues of the attacked plants. Specific defence mechanisms were shown to occur in cane portions close to the symptomatic ones, where JA- instead of SA- mediated signalling was up-regulated and active stilbenoids, such as viniferin, accumulated. It could be interesting to know if other grapevine cultivars showing low susceptibility to FD implement the same defence strategy as ‘Tocai friulano’.

Compartmentalization of FDp in symptomatic portions, and its absence in asymptomatic parts, indicated the possibility of recovery of ‘Tocai friulano’ plants. Further studies are in progress to verify the occurrence of recovery in the plants analyzed in the present work, through careful remotion of the canes with FD symptoms during pruning. These results indicated that agronomical practices to be adopted during FD management could depend on the grapevine cultivar.

In this work, for the first time, the mechanisms involved in successful defence against FD were highlighted in secondary phloem of different canes within infected ‘Tocai friulano’ plants, and this information could help us to understand more about strategies involved in recovery and its starting events. Moreover, the pathways investigated, and their spatial and temporal regulation, could suggest new genetic traits involved in the resistance against FD. The compartmentation of FDp by ‘Tocai friulano’ could be an interesting candidate character in a Quantitative Trait Locus investigation to identify the putative grapevine resistant genes against FD.

## Material and methods

### Vineyard description

The study was carried out in a vineyard located in Friuli Venezia Giulia (Northeastern Italy), in the province of Udine. The grapevine varieties cultivated in the vineyard are ‘Tocai friulano’ (1,5 ha) and ‘Pinot gris’ (1,80 ha). The grapevine plants were more than 25 years old and were grown with integrated pest management. The pruning system was the guyot method with two fruiting arms.

The vineyard was addressed due to the presence of ‘Tocai friulano’, which is scarcely susceptible to FD, planted close to ‘Pinot gris’, which is very susceptible. Moreover, during 2019 vegetative season there was a high population of *S. titanus*, verified by the positioning of yellow sticky traps from the middle of July to the middle of August in both varieties.

### Survey of FD symptoms and leaf diagnostic

All ‘Tocai friulano’ and ‘Pinot gris’ plants were usually surveyed in 2019, 2020 and 2021, and symptomatic plants were identified, mapped and labelled during the years.

In 2021, 20 plants were selected, which were asymptomatic in 2019-2020 and symptomatic in 2021: ten plants of ‘Tocai friulano’ and the next available neighbour ten plants of ‘Pinot gris’. The presence of FDp was evaluated by DNA extraction from symptomatic leaves followed by specific real-time PCR detection [47], and the genetic characterization at the 16S rRNA subgroup level was carried out by RFLP (Restriction Fragment Length Polymorphism) analyses as in Angelini et al. [48]. FD symptoms of the 20 plants were carefully observed in two time points: middle (July 2021) and end (September 2021) of the vegetative season. The FD-infected symptom evaluation was based on the plant’s subdivision in the following three classes of infection: (1)plants with less than half of FD symptomatic canes on one of the two 2-year-old arms; (2) plants with most of the canes showing FD symptoms on one of the two 2-year-old arms; (3) plant with both 2-year-old arms with FD symptomatic canes. Moreover, possible infection with the major grapevine viruses (*Grapevine fanleaf virus* (GFLV), *Arabis mosaic virus* (ArMV), *Grapevine leafroll associated virus-1, -2 and -3* (GRLaVs), *Grapevine virus A* (GVA), *Grapevine virus B* (GVB)) was determined as described in Bertazzon et al. [49]. The viral sanitary status of the plants was homogeneous: indeed, all plants were infected with GFLV and GLRaV-3 and tested negative for the presence of GRLaV-1, GRLaV-2, GVA, GVB and ArMV.

### Sampling of secondary phloem from 2-year-old arms and trunk

**Fig. 8:**
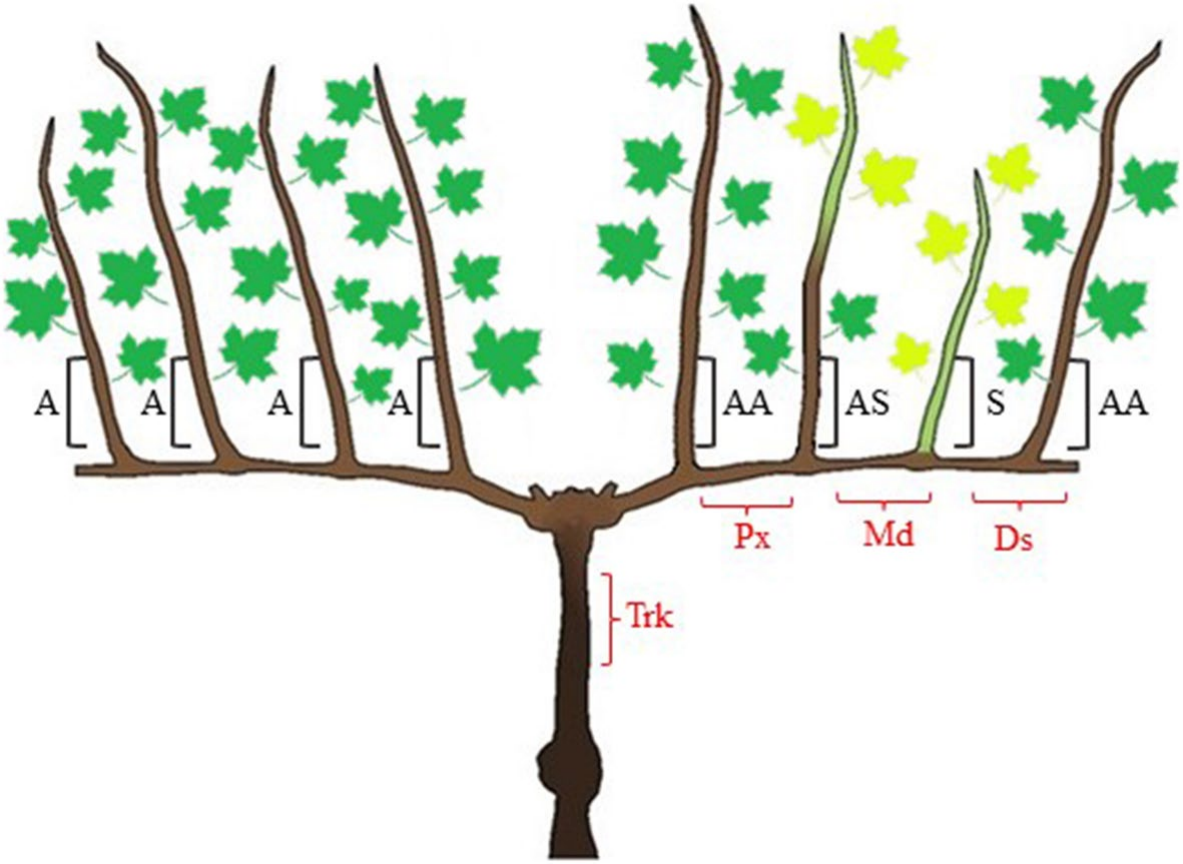
Schematic representation of the sampling portions collected in FD-infected ‘Tocai friulano’ and ‘Pinot gris’ plants. Samples collected in ‘Tocai friulano’ for gene expression analysis and evaluation of H_2_O_2_ and polyphenol contents are indicated in black. The 1-year-old canes grown from the symptomatic 2-year-old arm were distinguished in three theses: symptomless canes (AA), canes completely symptomatic from the base to the apex (S), and canes symptomatic in the apex and asymptomatic in the base (AS). The 1-year-old canes grown from the asymptomatic 2-year-old arm in symptomatic plants were completely symptomless and were identified as “A”. Samples collected for *Flavescence dorée* phytoplasma detection in 2-year-old arms and trunks of ‘Tocai friulano’ and ‘Pinot gris’ *Flavescence dorée*-infected plants are indicated in red. Three parts of the symptomatic 2-year-old arm (proximal (Px), median (Md), distal (Ds)) and one trunk section (Trk) per plant were collected. The symptomatic and asymptomatic leaves are indicated with yellow and green, respectively, while lignified and non-lignified 1-year-old canes are coloured in brown and green, respectively

Five plants of each variety were selected from the 1^st^ and 2^nd^ class of infection previously described, to study the distribution of FDp through the secondary phloem of 2-year-old arms and trunk in July and November. The sampling took place in vineyard using liquid nitrogen to ensure tissue preservation until the storing at -80°C. Three parts of the symptomatic 2-year-old arm (proximal, median, distal) and one trunk section per plant were collected for the molecular analyses (Fig. 8). The samples were obtained by cortical scraping of the arm/trunk longitudinal section (2 cm) after bark removal, and then were processed as described subsequently.

### Sampling of secondary phloem from basal portions of 1-year-old canes

A variable number of healthy and FD-infected plants (from 5 to 25) of the cultivar ‘Tocai friulano’ were sampled for two years (2020 and 2021), in September and November, corresponding to the end of the vegetative season and the dormant phase, respectively. The sample collection was performed to evaluate gene expression and polyphenol content on basal portions of 1-year-old canes growing in FD-infected plants. The pruning system with two fruiting arms per plant allowed the identification of plants with the coexistence of symptomatic and asymptomatic 2-year-old arms. The basal portions of 1-year-old canes from FD-infected plants were sampled according to the theses represented in Fig. 8. The 1-year-old canes grown from the symptomatic 2-year-old arms were distinguished in three theses: symptomless canes (AA), canes completely symptomatic from the base to the apex (S), and canes symptomatic in the apex and asymptomatic in the base (AS). The 1-year-old canes grown from the asymptomatic 2-year-old arm in symptomatic plants were completely symptomless and were identified as “A”. Samples were also collected from the basal portion of 1-year-old canes in healthy plants and were identified as “H”. Samples for molecular and metabolic analyses were obtained by longitudinally cortical scraping of canes (10 cm), after bark removal. The sampling took place in vineyard using liquid nitrogen to ensure tissue preservation until the storing at -80°C. The total number of collected samples for each thesis is reported in Table 1. Due to the few symptomatic plants, a small number of samples, not always representative of all the theses (Fig. 8), was collected in 2020. On the opposite, the increased occurrence of infected plants in 2021 made it possible to significantly increase the number of samples for each thesis. The leaves located in the basal portions of 1-year-old canes were collected and analysed to verify the presence or absence of FDp in the symptomatic (S), asymptomatic (AA, A, H) and in partially asymptomatic canes (AS) as described subsequently.

### RNA extraction, wood diagnosis and expression analysis of selected genes

Both the gene expression analyses and the study of the distribution of the phytoplasmas in the wood tissue were carried out on total RNA extracted samples. Samples of secondary phloem (100 mg) were homogenized in liquid nitrogen, total RNA was extracted using the RNeasy Plant Mini Kit (Qiagen) with a modified protocol as reported in Bertazzon et al. [49] and resuspended in RNAse-free water. The samples collected from leaves (50 mg) were homogenized in liquid nitrogen, total RNA was extracted using Norgen Plant /Fungi total RNA Purification kit and resuspended in the elution buffer provided with the kit. One unit of RNase-free DNase I (Invitrogen) was added to 1 µg of each extracted RNA to remove DNA residuals, incubated at 37 °C for 45 min, and 1 µL of 25 mM EDTA was added to stop the reaction. Subsequently, RNA was denatured at 95 °C for 5 minutes, then reverse transcribed with 200 units of Moloney Murine Leukemia Virus reverse transcriptase (Invitrogen) and random hexamer primers (Roche) at 42 °C for 50 min to obtain 50 µL of cDNA.

Real-time PCR assays were performed in 96-well plates on a Bio-Rad thermal cycler (model CFX 96), using the 2× Platinum SYBR Green qPCR Supermix UDG (Invitrogen) [50]. Each PCR reaction contained 0.3 µM of each primer and 1 µL of cDNA in a total volume of 10 µL, and was performed at least in duplicate. The PCR protocol was preceded by 3 min at 50 ºC (decontamination step) to allow optimal enzymatic activity of UDG (Uracyl DNA Glycosylase) and followed by 3 min at 95 ºC for activation of the Platinum Taq polymerase, deactivation of the UDG and denaturation of the cDNA sample. Afterwards, a two-step thermal protocol was carried out, consisting of 50 cycles of denaturation at 95 ºC for 5 s and annealing/extension at 60 ºC for 30 s. The same cycling conditions were employed for all the targets. Primers used for the identification of the presence of FDp were those reported in Angelini et al. [47]. Oligonucleotides used in real-time PCR experiments for detecting gene expression are described in Additional file 5. The gene specific primers were selected from the literature or were designed using Primer-BLAST software with the gene sequences obtained from the GenBank of the National Center for Biotechnology Information. The performance of each qPCR assay was evaluated by generating three standard curves over a broad range of cDNA dilutions (from undiluted to 10^-4^) with the obtainment of amplification efficiencies higher than 96% in all cases. The reference genes used to calculate the relative expression of selected genes were *glyceraldehyde-3-phosphate dehydrogenase (GAPDH)* and *cytochrome c oxidase (COX)*; the last one was used also to normalize the FDp titre. Data were statistically analysed with one-way ANOVA test using Student–Newman–Keuls post hoc test.

### Quantification of stilbenes and polyphenols content

The analyses were performed on the same samples collected for gene expression study in November 2020, and in September and November 2021 (Table 1). The material obtained by the cortical scraping of the basal portion of 1-year-old canes (250 mg) was collected, grounded with liquid nitrogen and then stored at -20°C until processing. The stilbene extraction was performed according to the protocol described by Rayne et al. [50] and stilbene analysis was carried out as described in Vincenzi et al. [51]. The same extracts were also analysed in HPLC for identification and quantification of polyphenolic compounds, by injection on a Kinetex C18 column (150 x 4.6 mm, Phenomenex) using a gradient of methanol + 0.1% trifluoroacetic acid (TFA) in water + 0.1% TFA. The quantification was performed using calibration curves of pure commercial standards. The detection was performed at 280 nm.

### Determination of H_2_O_2_ content

The analysis was performed on the same samples collected for gene expression study in November 2020 (Table 1). The peroxidase-coupled assay (3-dimethylaminobenzoic acid [DMAB]–3-methyl-2-benzothiazolinone hydrazone [MBTH]–POX) method was adapted to evaluate the H_2_O_2_ content in the samples obtained by cortical scraping of the basal portion of 1-year-old canes. The material collected in liquid nitrogen (100 mg) was stored at -20°C and grounded with liquid nitrogen. The H_2_O_2_ analysis was conducted as described previously [19, 52]. Data were statistically analysed with one-way ANOVA test using Student–Newman–Keuls post hoc test.

## Supplementary Information

**Additional file 1: **Relative quantification of FD phytoplasma, expressed as arbitrary units (log2), in 2-year-old arms and trunk of symptomatic plants of 'Pinot gris'. The portions of 2-year-old fruit arm, from which symptomatic canes grew, are indicated in bold. The gene COX was used for normalization.

**Additional file 2: **log2 relative transcript level of selected categories of genes obtained from 1-year-old canes grown on symptomatic (S, AS and AA) and asymptomatic (A) 2-year-old arms of Flavescence dorée infected 'Tocai friulano' plants or from healthy plants (H). For each gene the relative expression was calculated by setting a value of 1 as the lowest value among S, AS, AA, A or H samples. Samples were collected for two years (2020 and 2021) during September and November. Normalisation of transcriptional levels were performed using GAPDH and COX as endogenous controls. Significant differences (P ≤ 0.05) among treatments are indicated by different letters, according to parametric one-way ANOVA using Student Newman Keuls test.

**Additional file 3: **Flavonoids content: A) proanthocyanidins; B) catechins; C) epicatechins. Samples were collected from 1-year-old canes grown on symptomatic (S, AS and AA) and asymptomatic (A) 2-year-old arms of Flavescence dorée infected 'Tocai friulano' plants in September (2021) and November (2020 and 2021), and from healthy plants (H) in November (2020). Significant differences (P ≤ 0.05) among treatments are indicated by different letters, according to parametric one-way ANOVA using Student Newman Keuls test.

**Additional file 4: **Hydroxigen peroxide (H_2_O_2_) content. Samples were collected from 1-year-old canes growth from the symptomatic (S and AA) and asymptomatic (A) 2-year-old arms of Flavescence dorée infected 'Tocai friulano' plants and from healthy plants (H) in November (2020). Significant differences (P ≤ 0.05) among treatments are indicated by different letters, according to parametric one-way ANOVA using Student Newman Keuls test.

**Additional file 5: **The gene specific primers were selected from the literature or were designed using Primer-BLAST software with the gene sequences obtained from the GenBank of the National Center for Biotechnology Information. NP: accession not provided.

## Data Availability

All data generated or analysed during this study are included in this published article and its supplementary information files.

## Abbreviations

A: Asymptomatic cane growing from asymptomatic 2-year-old arm of FD- infected plants
AA: Asymptomatic cane growing from symptomatic 2-year-old arm of FD- infected plants
ACS: 1-aminocyclopropane-1-carboxylate synthase-like
ANOVA: Analysis of variance
AOS: Allene oxide synthase
APX: Ascorbate peroxidase
ArMV: Arabis mosaic virus
AS: Canes symptomatic in the apex and asymptomatic in the base, growing from symptomatic 2-year-old arm of FD- infected plants
BN: *Bois noir*
BNp: *Bois noir* phytoplasma
CAS: Callose synthases
CAT: Catalase
cDNA: Complementary DNA
COX: Cytochrome c oxidase
DMAB: Dimethylaminobenzoic acid
DMR: Downy mildew resistance
dT: Deoxythymine
EDTA: Ethylenediaminetetraacetic acid
Et: Ethylene
FD: *Flavescence dorée*
FDp: *Flavescence dorée* phytoplasma
GAPDH: Glyceraldehyde-3-phosphate dehydrogenase
GFLV: Grapevine fanleaf virus
GLP: Germin-like protein
GOX: Glycolate oxidase
GPX4: Glutathione peroxidase
GRLaV: Grapevine leafroll associated virus
GVA: Grapevine virus A
GVB: Grapevine virus B
GY: Grapevine Yellows
H: Healthy cane grown from healthy plants
H_2_O_2_: Hydrogen peroxide
HPLC: High performance liquid cromatography
JA: Jasmonic acid
JAR: Jasmonate resistant
LOX: Lipoxygenase
MBTH: methyl-benzothiazolinone hydrazone
NPR: Nonexpressor of pathogenesis-related genes
‘Pinot gris’: Pinot gris
PCR: Polymerase Chain Reaction
PER: Peroxidase
POX: Phenoloxidase
PR proteins: Pathogenesis related proteins
qRT-PCR: Quantitative Reverse Transcriptase - Polymerase Chain Reaction
S: Symptomatic cane
SA: Salicylic acid
STS: Stilbene synthase
‘Tocai friulano’: Tocai friulano
TFA: trifluoroacetic acid
UDG: Uracyl DNA glycosylase
