# Supplementary material for: A successful defence strategy in grapevine cultivar ‘Tocai friulano’ provides compartmentation of grapevine Flavescence dorée phytoplasma
Source: BMC Plant Biol. 2023 Mar 25;23:161. doi: 10.1186/s12870-023-04122-0 (PMC10039607; doi:10.1186/s12870-023-04122-0)
Supplement: Supplementary file 5 — Additional file 5: The gene specific primers were selected from the literature or were designed using Primer-BLAST software with the gene sequences obtained from the GenBank of the National Center for Biotechnology Information. NP: accession not provided. [file 12870_2023_4122_MOESM5_ESM.docx]

**Additional file 5:** The gene specific primers were selected from the literature or were designed using Primer-BLAST software with the gene sequences obtained from the GenBank of the National Center for Biotechnology Information. NP: accession not provided

|  | **short name** | **NCBI accession number** | **Gene description** | **Primer sequence 5′–3′ forward** | **Primer sequence 5′–3′ reverse** | **References** |
| --- | --- | --- | --- | --- | --- | --- |
| **H_2_O_2_ production** | **GOX** | XM_002278068 | Glycolate oxidase | GTGGTTGCCGCTCCCTTA | AGCGTCCCAGTCAGTCATGAT | (1) |
|  | **GLP1** | EF064171 | Germin-like protein | AAGAATGCTGTGTTTGTAAATGGAAA | TCGGCGACGGTCAGGTT | (1) |
|  | **GLP3** | AY298727 | Germin-like protein | AGCAAATGCAGTCTTTGGATCA | GGCCCTGGTGAGAACATCA | (1) |
| **H_2_O_2_ scavenging** | **CAT1** | XM_002270667 | Catalase | CGCCCTTCGAGTGCTTACA | CAGGAGCACCGGAAT | (1) |
|  | **APX3** | XM_002278245 | Ascorbate peroxidase | CCCAATCCCCAGACCAATC | TCGGCGTCTGCTGAGCTT | (1) |
|  | **APX6** | XM_002282641 | Ascorbate peroxidase | GCCCACTCTCCCCATTCTC | TGGAGTTTTGGCGGGAAAT | (1) |
|  | **GPX4** | VIT_07s0104g00320 | Glutathione peroxidase 4 | CGTGTAAATGGCCCTGATGCT | CCAAGAAATCCACTTTTGTGTGCTT | (2) |
| **Leaf markers of recovery** | **GDSL1** | VIT_09s0002g00540 | Lipase GDSL 1 | CTCAGAACCATGTGGCCTTT | CATCGGAAAACCTCCCAGTA | This work |
|  | **WRKY2** | NM_001281181 | WRKY Transcription factor 2 | AAGTATGGGCAGAAAGTTGTC | CTGTTGGCTGTGTTGTGG | (3) |
| **Cell wall reinforcement** | **PER** | XM_002274762.1 | Lignin-forming peroxidase | TAAGCGCCACAAGAACACTG | GGACCTCCTTGTTGAGTCCA | (4) |
|  | **CAS** | AJ430780 | Callose synthase | ACATGGCATTCGTCCTCCTACC | GGCCTTGCTGATACCACCTCG | (5) |
| **Phenolic pathway** | **STS1** | NP | Stilbene synthase 1 | TACGCCAAGAGATTATCACT | CTAAAGAGTCCAAAGCATCT | (6) |
|  | **STS16** | NP | Stilbene synthase 16 | CTTTTGACCCAATTGGAATCAAC | TGACATGTTCCCATATTCACTTAG | (7) |
|  | **STS48** | NP | Stilbene synthase 48 | CTTGAAGGGGGAAAATGCT | TTACTGCATTGAAGGGTAAACC | (7) |
| **SA-signalling and SA-responsive genes** | **NPR1** | VIT_11s0016g01990 | Regulatory protein NPR1-like | GGCGGTTTTGGGGTATTTGT | AGAGCACCTCCACCATGAAA | (8) |
|  | **PR1** | NP | PR1 (unknown function) | ATGGGCTTGTTTAAGATTTCACT | GTTCCATGTCATAGACCCAACACCAACTTG | (9) |
|  | **PR2** | XM_002277475 | Beta-1,3-glucanase | CAACTTGCCACCGCCAGGGC | AGGGCTTGGAGAGCAGCTTGG | (4) |
|  | **PR5** | XM_002282928.1 | Thaumatin-like/Osmotin | GGAGGCAATGGTTTCCACCTTGGG | ACTTGGACGGGACCATAGAGGTTAG | (4) |
|  | **WRKY70** | VIT_13s0067g03140 | WRKY transcription factor 70 | CCCAGTTCATTGGAAGCTC | CCCTCTTTGAGTAACCATTGC | (10) |
|  | **DMR6** | VIT_16s0098g00860 | DMR6-like oxygenase | GCAGGCTCTATGGTTTTTTCC | GCTTCATCTTCTCCTCCACC | (2) |
| **JA-signalling and JA-responsive genes** | **LOX1** | VIT_06s0004g01510 | 13-lypoxygenase | ACTGCCCCGACCTTCTTC | AGCCAACCCTAACATTCCTG | (11) |
|  | **LOX2** | VIT_13s0064g01480 | 13-lypoxygenase | AGTGGAGACGTCGAAGGAGA | GCGCTAACAAGCTCCAAAAG | (10) |
|  | **LOX3** | VIT_09s0002g01080 | 13-lypoxygenase | TGGGAAACCCTGATAATGGA | ATTGGAAATGGCAGCTCAAC | (10) |
|  | **AOS** | VIT_03s0063g01820 | Allene oxide synthase | GTCCTCCTCGACTCCATCAG | TCGGAAGGGTCGAGATATGC | (8) |
|  | **JAR** | XM_002280702.1 | Jasmonate-resistant 1 | CCGAAGTGCTGGCCCCAGAG | AACGCTCACTTCGCCGCTGA | (4) |
|  | **PR4** | XM_002264684.1 | Chitin binding Chitinases type I, II | CCCAGAGCGCCAGCAATGTGA | TTGCTGCGCCATGCCAAGGG | (4) |
|  | **PR6** | VIT_00s0270g00120 | Alpha-amylase/subtilisin inhibitor-like | TCCGGACAGGCTCTTCAAC | CCCTGATAATCGTCTCTTGCTC | (10) |
|  | **PR10** | AJ291705 | Ribonuclease-like | GCTCAAAGTGGTGGCTTCTC | CTCTACATCGCCCTTGGTGT | (4) |
|  | **PR12** | XM_010666410.2 | Defensin | TCCCATGTGTGTCTGCATTT | TCCCGGTTTGTGATTATGGT | This work |
| **Et-signalling** | **ACS** | VIT_02s0025g00360 | 1-aminocyclopropane-1-carboxylate synthase-like | GCTCATCCTTCCATTGCTCG | TGGCAGACACCTCCTTTTCT | (8) |

1. Gambino G, Boccacci P, Margaria P, Palmano S, Gribaudo I. Hydrogen Peroxide Accumulation and Transcriptional Changes in Grapevines Recovered from Flavescence Dorée Disease. Phytopathology. 2013;103(8):776–84.

2. Prezelj N, Covington E, Roitsch T, Gruden K, Fragner L, Weckwerth W, et al. Metabolic Consequences of Infection of Grapevine (Vitis vinifera L.) cv. «Modra frankinja» with Flavescence Dorée Phytoplasma. Front Plant Sci. 2016;7:711.

3. Paolacci AR, Catarcione G, Ederli L, Zadra C, Pasqualini S, Badiani M, et al. Jasmonate-mediated defence responses, unlike salicylate-mediated responses, are involved in the recovery of grapevine from bois noir disease. BMC Plant Biol. 2017;17(1):118.

4. Dufour MC, Magnin N, Dumas B, Vergnes S, Corio-Costet MF. High-throughput gene-expression quantification of grapevine defense responses in the field using microfluidic dynamic arrays. BMC Genomics. 2016;17:957.

5. Repetto O, Bertazzon N, De Rosso M, Miotti L, Flamini R, Angelini E, et al. Low susceptibility of grapevine infected by GLRaV-3 to late Plasmopara viticola infections: Towards understanding the phenomenon. Physiological and Molecular Plant Pathology. 2012;79:55–63.

6. Aziz A, Poinssot B, Daire X, Adrian M, Bézier A, Lambert B, et al. Laminarin Elicits Defense Responses in Grapevine and Induces Protection Against Botrytis cinerea and Plasmopara viticola. MPMI. 2003;16(12):1118–28.

7. Vannozzi A, Dry IB, Fasoli M, Zenoni S, Lucchin M. Genome-wide analysis of the grapevine stilbene synthase multigenic family: genomic organization and expression profiles upon biotic and abiotic stresses. BMC Plant Biol. 2012;12:130.

8. Shangguan L, Mu Q, Fang X, Zhang K, Jia H, Li X, et al. RNA-Sequencing Reveals Biological Networks during Table Grapevine (‘Fujiminori’) Fruit Development. PLOS ONE. 2017;12(1):e0170571.

9. Li ZT, Dhekney SA, Gray DJ. PR-1 gene family of grapevine: a uniquely duplicated PR-1 gene from a Vitis interspecific hybrid confers high level resistance to bacterial disease in transgenic tobacco. Plant Cell Rep. 2011;30(1):1–11.

10. Bertazzon N, Bagnaresi P, Forte V, Mazzucotelli E, Filippin L, Guerra D, et al. Grapevine comparative early transcriptomic profiling suggests that Flavescence dorée phytoplasma represses plant responses induced by vector feeding in susceptible varieties. BMC Genomics. 2019;20(1):526.

11. Pilati S, Brazzale D, Guella G, Milli A, Ruberti C, Biasioli F, et al. The onset of grapevine berry ripening is characterized by ROS accumulation and lipoxygenase-mediated membrane peroxidation in the skin. BMC Plant Biology. 2014;14(1):87.
